# Non-invasive biomarkers in assisted reproductive technologies, current evidence, clinical utility and future perspectives

**DOI:** 10.3389/frph.2026.1875776

**Published:** 2026-07-02

**Authors:** Athanasios Zikopoulos, Athanasia Sesse, Periklis Katopodis, Ariadni Theodora Katopodi, Vasilis Sebastian Paraschos, Maria Filiponi, Ioanna Bouba, Charilaos Kostoulas, Athanasios Zachariou, Ioannis Georgiou

**Affiliations:** 1 Laboratory of Medical Genetics in Clinical Practice, Faculty of Medicine, School of Health Sciences, University of Ioannina, Ioannina, Greece; 2 Department of Obstetrics and Gynecology, Corewell Health Hospital, Dearborn, MI, United States; 3 Department of Urology, Faculty of Medicine, School of Health Sciences, University of Ioannina, Ioannina, Greece

**Keywords:** artificial intelligence in IVF, assisted reproductive technologies, embryo selection, endometrial receptivity, follicular fluid, *in vitro* fertilization, non-invasive biomarkers, omic technologies

## Abstract

Although assisted reproductive technologies have significantly advanced the treatment of infertility, overall success rates remain limited by the lack of accurate and safe tools for assessing reproductive potential. Conventional embryo selection and endometrial evaluation rely largely on morphological and non-functional criteria, which provide only indirect insight into the underlying biological competence of the embryo oocyte and uterine environment. In this context the development of non-invasive biomarkers has emerged as a critical objective in modern reproductive medicine. This review discusses current evidence on non-invasive biomarkers in *in vitro* fertilization and assisted reproductive technologies with emphasis on embryo derived follicular fluid and endometrial indicators. Advances in time lapse imaging analysis of spent embryo culture media follicular fluid profiling and uterine fluid assessment are discussed in relation to their biological significance and association with clinical outcomes. The role of metabolomic proteomic transcriptomic and genetic signals is examined alongside emerging applications of artificial intelligence for data integration and outcome prediction. While numerous non-invasive biomarkers demonstrate promising associations with embryo viability implantation potential and surrogate pregnancy outcomes, with limited evidence in live birth rates, their clinical translation is constrained by methodological heterogeneity, limited validation, and challenges in standardization. Integrative multi parameter approaches rather than single biomarker strategies appear most likely to enhance predictive accuracy. Continued efforts toward data harmonization, large scale validation, and clinically oriented model development are essential for the successful incorporation of non-invasive biomarkers into routine IVF practice. Curated databases and relevant AI applications may facilitate and help progress non-invasive biomarkers in ART.

## Introduction

Assisted reproductive technologies (ART) have transformed the management of infertility and now account for a substantial proportion of births worldwide (1). Despite continuous advances in laboratory techniques, culture systems and clinical protocols, overall success rates remain modest and highly variable among patients, facilities and countries. A major limiting factor in *in vitro* fertilization (IVF) outcomes is the inability to accurately and reproducibly assess embryo viability, oocyte competence and endometrial receptivity using methods that are both biologically informative and clinically safe. Current decision making in IVF still relies heavily on indirect morphological criteria and observations that are static, which often fail to capture the underlying molecular and metabolic status, or other functional parameters of the reproductive system (2, 3).

The pursuit of reliable biomarkers capable of predicting reproductive, developmental, and metabolic potential has therefore become a central issue in reproductive medicine. Traditional approaches to biomarker discovery have frequently involved invasive procedures such as embryo biopsy or endometrial sampling which although informative, introduce biological risk, ethical concerns and technical variability (4). These limitations have accelerated research interest in non-invasive biomarkers that can be obtained without compromising the embryo integrity or disrupting the physiological environment and pathways of the reproductive tract. Non-invasive strategies offer the potential to improve clinical outcomes while aligning with the ethical imperative of minimizing intervention in early human development in accordance to Oviedo ethical principles (5).

Recent advances in molecular biology, analytical chemistry, and computational sciences have enabled the detection of subtle biological signals in previously considered inert media and fluids (5, 6). Spent embryo culture media, follicular fluid uterine secretions, spectroscopy and time lapse imaging data, are now recognized as rich sources of information reflecting embryo metabolism cellular communication and developmental competence. In parallel developments in omic technologies, databases, and artificial intelligence (AI), have expanded the capacity to integrate complex datasets. These approaches enable the generation of predictive models that may outperform conventional assessment methods (7, 8).

This review aims to provide a comprehensive and critically balanced overview of non-invasive biomarkers in IVF and ART. Emphasis is focused on embryo derived, follicular and endometrial biomarkers, as well as on the technological platforms enabling their discovery and interpretation. Attention is given to translational relevance, distinguishing biomarkers that demonstrate consistent associations with clinical outcomes from those that remain exploratory. By synthesizing accumulated current evidence and highlighting remaining challenges this article seeks to clarify the role of non-invasive biomarkers in shaping the future of precision and effective reproductive care.

## Literature search strategy and review methodology

This review was conducted to summarize current evidence regarding non-invasive biomarkers in ART. A literature search was performed using PubMed and Scopus databases. Relevant articles published in English up to March 2026 were identified using combinations of keywords including “IVF”, “assisted reproductive technologies”, “non-invasive biomarkers”, “embryo selection”, “follicular fluid”, “endometrial receptivity”, “metabolomics”, “proteomics”, “microRNA”. “cell-free DNA”, and “artificial intelligence”. Studies focusing on invasive diagnostic approaches without relevance to non-invasive biomarker development were excluded. The selected literature was critically evaluated with emphasis on biological rationale, clinical applicability, validation status and translational potential.

## ART and the clinical need for non-invasive biomarkers

ART encompasses a sequence of highly regulated clinical and laboratory interventions designed to overcome infertility and optimize the probability of achieving a viable pregnancy. Standard IVF workflows include controlled ovarian stimulation, oocyte retrieval, IVF embryo culture and embryo transfer. At each stage clinical decisions are made that directly influence cumulative live birth rates, yet these decisions are frequently guided by indirect indicators of reproductive potential and they are not always objective or based on curated data. Parameters such as patient age, ovarian reserve markers (e.g., anti- Müllerian hormone levels and antral follicle count), fertilization rates and embryo morphology are routinely used as indirect indicators of reproductive potential. Although clinically valuable, these measures primarily reflect broad demographic, ovarian, or developmental characteristics and provide only partial insight into the complex biological processes that determine implantation and ongoing pregnancy (9, 10).

The assessment of embryo quality remains one of the most critical and challenging steps in IVF. Conventional morphological grading systems evaluate static features such as blastomere symmetry, fragmentation, and blastocyst expansion. While these criteria are easy to apply and widely adopted they exhibit limited predictive power for euploidy, metabolic fitness and implantation competence (10). Embryos with optimal morphology may fail to implant whereas morphologically suboptimal embryos can result in healthy live births (11). This discrepancy highlights the fundamental limitation of morphology based assessment in capturing the dynamic molecular and metabolic state of the developing embryo (12).

Invasive diagnostic techniques were introduced to overcome these shortcomings particularly preimplantation genetic testing which enables the detection of chromosomal abnormalities prior to transfer (4, 13). Highly informative embryo biopsy, involves physical manipulation of the embryo which requires specialized expertise and raises concerns regarding potential effects on embryonic development. In addition the invasive nature of these techniques limits their universal application and contributes to increased cost and procedural complexity (4). Furthermore, these approaches are affected by intrinsic biological limitations, particularly embryonic mosaicism, which may result in discordant genetic interpretations depending on sampling strategy and analytical resolution (14).

Similarly invasive procedures used for endometrial assessment, such as endometrial biopsy, are important for evaluating receptivity but are not easily repeatable and may alter the uterine environment when attempted (15).

Non-invasive biomarkers are put forward as an attractive alternative by providing biologically relevant information without direct tissue disruption (5). These biomarkers may reflect embryo metabolism, cellular stress, mitochondrial activity, immune signalling, apoptosis or endometrial readiness through analytes released into surrounding fluids or captured through imaging based technologies (7). Importantly non-invasive approaches allow longitudinal assessment and repeated measurements enabling dynamic monitoring rather than single time point evaluation (16).

The clinical demand for non-invasive biomarkers in medicine is further driven by ethical considerations and patient expectations. As single embryo transfer becomes standard practice in many regions, accurate embryo selection is essential to maintain high success rates while minimizing multiple pregnancies (17). Non-invasive biomarkers have the potential to support this paradigm by refining selection strategies and reducing reliance on invasive testing. Collectively these factors underscore the urgent need for robust non-invasive biomarkers that can be seamlessly integrated into routine ART workflows and reliably inform clinical decision making.

### Embryo derived non-invasive biomarkers in IVF

The preimplantation embryo is a dynamic biological system whose developmental potential is governed by timely regulated metabolic, genetic and epigenetic processes. As direct interrogation of embryonic tissues is inherently limited in clinical practice, considerable effort has been directed toward identifying indirect signals that accurately reflect embryo competence. Among all non-invasive approaches embryo derived biomarkers have attracted particular attention as they provide information at the most decisive stage of IVF namely embryo selection prior to transfer (18, 19).

Time lapse imaging represents one of the earliest and most widely adopted non-invasive technologies in contemporary IVF laboratories. Continuous monitoring of embryo development enables the extraction of morphokinetic parameters such as cleavage timing, cell cycle synchrony, and blastocyst formation dynamics (20). These parameters capture aspects of developmental temporal stages that are not observable through static morphology alone. Several studies have demonstrated associations between specific morphokinetic patterns and implantation potential suggesting that developmental timing reflects underlying cellular organization and genomic stability, or genomic surveillance (21, 22). Nevertheless, the clinical performance of time lapse-based algorithms is influenced by variability in culture conditions, laboratory protocols, and ambryo culture systems. Importantly, recent large randomized studies suggest that time-lapse imaging alone does not necessarily improve live birth outcomes, indicating that morphokinetic assessment should be regarded as a supportive tool rather than a standalone strategy for embryo selection (23). Consistent with these findings, Armstrong et al. concluded that the available evidence was insufficient to demonstrate a clear clinical benefit of time-lapse systems compared with conventional embryo incubation and assessment methods (24). More recent randomized clinical trials have further highlighted the need to evaluate uninterrupted embryo culture and time-lapse-based embryo selection as distinct components, as improvements in culture conditions do not necessarily translate into enhanced embryo selection performance (25).

Beyond imaging, the biochemical composition of spent embryo culture media has emerged as a rich source of molecular information. Developing embryos actively consume and secrete metabolites proteins and nucleic acids into their surrounding environment. Metabolomic profiling of culture media has revealed differences in amino acid turnover energy substrate utilization and oxidative stress related metabolites between embryos with divergent developmental outcomes. These metabolic fingerprints are thought to mirror mitochondrial efficiency redox balance and overall cellular fitness. While promising, the sensitivity of metabolomic approaches is influenced by media composition embryo number and analytical platforms requiring rigorous methodological control (7, 26). Although these findings are promising, the clinical translation of spent culture media profiling remains constrained by media-related background signals, platform-specific variability, and the lack of universally accepted predictive cutoffs (27).

Metabolomic and proteomic analyses are additionally influenced by pre-analytical and analytical variability, including differences in culture media composition, sample handling, and storage conditions. Instrument-specific biases, batch effects, and lack of standardized normalization strategies across platforms further contribute to inter-laboratory variability and limit reproducibility.

Proteomic analysis of spent culture media has further expanded the repertoire of potential embryo derived biomarkers. Secreted proteins involved in cell adhesion, stress response and growth signalling have been correlated with blastocyst quality and implantation success (6, 28). Similarly extracellular microRNAs released by embryos are increasingly recognized as stable and biologically meaningful indicators of embryonic gene regulation (29). MicroRNA profiles in culture media appear to reflect early developmental programming and may provide insight into embryo viability beyond chromosomal status alone (30, 31). However most proteomic and microRNA studies remain exploratory and large-scale prospective validation is still lacking.

A particularly evolving area is the analysis of cell free DNA released into spent embryo culture media as the basis for non-invasive preimplantation genetic testing. This approach aims to infer chromosomal status without embryo biopsy by analysing embryonic DNA fragments present in the culture environment. Initial reports have demonstrated moderate concordance with conventional trophectoderm biopsy suggesting potential clinical utility (32, 33).

A major limitation of cell free DNA-based approaches is the risk of contamination from maternal or exogenous DNA sources, which may compromise analytical specificity. In addition, variability in DNA yield, amplification efficiency, and sequencing depth across laboratories further affects reproducibility. Differences in analytical platforms and bioinformatic pipelines also contribute to inter-study heterogeneity, limiting the robustness of clinical implementation. At the same time technical challenges including DNA contamination, mosaicism and variable DNA yield have raised concerns regarding reliability. As a result, non-invasive genetic testing remains an adjunct rather than a replacement for established methods (34, 35).

Collectively, embryo derived non-invasive biomarkers offer a multifaceted view of embryonic health encompassing developmental kinetics metabolic activity and molecular signalling. While none of these approaches alone has yet achieved sufficient accuracy to serve as a standalone diagnostic tool, their combined use may enable more refined embryo selection strategies. The integration of multiple embryo derived signals represents a critical step toward improving IVF outcomes while preserving embryo integrity (36).

### Follicular fluid and oocyte related non-invasive biomarkers

Oocyte competence is established well before fertilization and is profoundly influenced by the biochemical and cellular environment of the ovarian follicle. Follicular fluid represents the immediate microenvironment of the developing oocyte and contains a complex mixture of hormones, metabolites, growth factors, cytokines and extracellular vesicles derived from both the oocyte and surrounding somatic cells (37). As such it provides a valuable source of non-invasive biomarkers that reflect oocyte quality and developmental potential, without direct manipulation of the gamete itself (38, 39). Among these candidates, certain hormonal and lipidomic markers have shown associations with oocyte competence and IVF outcomes; however, their predictive performance is often inconsistent across stimulation protocols and patient populations, which limits immediate clinical applicability (40–42).

Hormonal composition of follicular fluid has been extensively studied in relation to oocyte maturation and fertilization capacity. Concentrations of oestradiol, progesterone and androgens within individual follicles appear to influence meiotic progression and cytoplasmic maturation (40, 41). In parallel metabolic profiling has identified associations between glucose, lactate, amino acids, and lipid metabolites and subsequent embryo development. These metabolic signatures are thought to represent the energetic demands of the oocyte and the efficiency of metabolic coupling between the oocyte and the granulosa cells (43, 44).

Oxidative stress is another critical determinant of oocyte competence and follicular fluid as it provides indirect insight into the redox state of the follicular environment (45). Biomarkers related to reactive oxygen species, antioxidant capacity and lipid peroxidation have been correlated with fertilization rates and embryo quality (46). An imbalance favouring oxidative stress may impair mitochondrial function, disrupt spindle formation and compromise chromosomal segregation. Mitochondrial related markers, including mitochondrial DNA content and metabolites linked to oxidative phosphorylation have therefore gained attention as indicators of oocyte bioenergetic health (47, 48).

Inflammatory and immune mediators within follicular fluid, further modulate oocyte development and competence. Cytokines, chemokines and growth factors contribute to follicular remodelling ovulation and cumulus oocyte complex expansion (49). Altered inflammatory profiles have been associated with poor oocyte quality and reduced implantation rates particularly in conditions such as polycystic ovary syndrome and advanced maternal age. These findings highlight the interconnected nature of metabolic oxidative and immune pathways within the follicular niche (38, 50, 51).

Despite their biological relevance, interpretation of follicular fluid biomarkers remains challenging because of inter-follicular variability and methodological heterogeneity. Nevertheless, when evaluated in conjunction with embryo derived markers, follicular fluid analysis holds significant promise for enhancing individualized assessment of reproductive potential and refining ART decision making (37, 42).

### Endometrial and uterine microenvironment biomarkers

Successful implantation requires precise synchronization between a developmentally competent embryo and a receptive endometrium. While embryo quality has traditionally been the primary focus of IVF optimization increasing evidence indicates that uterine factors play an equally decisive role in determining clinical outcomes. The endometrial and uterine microenvironment undergoes dynamic molecular and structural changes during the implantation window and non-invasive access to this information is of particular clinical interest (37, 52).

Uterine fluid has emerged as a promising medium for assessing endometrial receptivity without the need for tissue biopsy (53–55). This fluid contains proteins, cytokines, metabolites and nucleic acids secreted by endometrial epithelial and stromal cells and reflects the functional state of the endometrium. Proteomic analyses have identified differential expression of adhesion molecules, growth factors and immune mediators associated with implantation success. These molecules are involved in embryo attachment, immune tolerance and decidualization processes critical for early pregnancy establishment (53, 56). However, despite their biological plausibility, most endometrial receptivity assays and uterine fluid biomarkers have not yet demonstrated sufficient reproducibility or prospective clinical benefit to support routine implementation in IVF practice.

Transcriptomic profiling of uterine fluid has further expanded understanding of endometrial receptivity. Messenger RNA and microRNA signatures detected in uterine secretions appear to reflect endometrial gene expression patterns associated with the receptive phase. Such profiles may provide temporal information regarding the implantation window and help identify subtle asynchrony between embryo development and endometrial readiness (57). Importantly, non-invasive transcriptomic approaches offer the potential for repeated assessment across cycles enabling more personalized timing of embryo transfer (54, 55).

The uterine microbiome has also gained attention as a non-invasive biomarker of implantation potential. Alterations in microbial composition, particularly reduced dominance of Lactobacillus species, have been linked to impaired implantation and increased pregnancy loss. Microbiome derived metabolites and immune interactions may influence endometrial inflammation and receptivity. Although causal relationships remain under investigation these findings underscore the importance of the uterine ecosystem in reproductive success (58, 59).

Despite their promising clinical value in precision medicine, endometrial and uterine biomarkers face challenges related to sample collection, timing, analytical sensitivity and inter individual variability. Nonetheless, non-invasive assessment of the uterine environment represents a critical complement to embryo focused strategies and contributes to a more holistic understanding of implantation failure in IVF (52, 54).

The principal categories of non-invasive biomarkers currently investigated in ART, together with their proposed clinical applications and current level of evidence, are summarized in Table 1.

**Table 1:** Main categories of non-invasive biomarkers investigated in ART, their biological source, proposed clinical applications, and current challenges for clinical implementation.

Biomarker category	Sample/source	Proposed clinical application	Major challenges
Morphokinetic parameters	Time-lapse imaging	Embryo selection	Inter-laboratory variability
Cell-free DNA	Spent culture medium	Non-invasive aneuploidy screening	Mosaicism contamination
Extracellular microRNAs	Spent culture medium	Embryo competence assessment	Limited validation
Metabolomic signatures	Spent culture medium	Implantation prediction	Methodological heterogeneity
Hormonal/metabolic markers	Follicular fluid	Oocyte competence assessment	Follicular variability
Cytokines and inflammatory mediators	Follicular fluid	Reproductive outcome prediction	Lack of standardization
Transcriptomic biomarkers	Uterine fluid	Endometrial receptivity assessment	Small validation cohorts
Endometrial microbiome	Uterine environment	Implantation prediction	Methodological variability
AI-integrated models	Multi-model datasets	Embryo selection and outcome prediction	External validation

## Omic technologies and AI in biomarker integration

The increasing complexity of non-invasive biomarker data in IVF has necessitated the adoption of advanced analytical frameworks capable of integrating heterogeneous biological signals. Omics technologies including metabolomics, proteomics, and transcriptomics have enabled high resolution profiling of reproductive fluids and culture environments. These approaches reveal multilayered molecular patterns associated with reproductive success. While each omic approach offers valuable insights their greatest analytical potential emerges when they are combined. Integrated multi-omics analysis more accurately reflects the multifactorial nature of embryo development and implantation (54, 60).

Multi omic strategies aim to capture the interaction between metabolic activity, protein signalling and gene regulation within the reproductive system. In the context of IVF this integrative approach allows simultaneous evaluation of embryo derived signals, follicular environment characteristics and endometrial receptivity markers. Such comprehensive profiling has revealed coordinated molecular networks underlying oocyte competence, embryo viability and implantation potential. However the high dimensionality of omic datasets presents challenges related to data interpretation reproducibility and clinical scalability (54, 61, 62). At present, multi-omic and AI-based approaches are best considered decision-support tools that may complement, but should not replace, established embryological and clinical assessment until robust external validation is available (8, 36).

AI and machine learning methods have emerged as essential tools for extracting clinically meaningful information from complex biomarker datasets. Supervised and unsupervised algorithms can identify subtle patterns and nonlinear relationships that are not apparent through conventional statistical analysis. In IVF applications, AI driven models have been developed to predict embryo implantation potential, pregnancy outcomes and cumulative live birth rates using combinations of imaging data molecular biomarkers and clinical parameters. These models hold promise for supporting clinician decision making by providing objective data driven assessments (63, 64). A landmark example is the STORK framework described by Khosravi et al., which used deep learning to classify human blastocyst quality from embryo images and demonstrated high discrimination performance (65). More recently, Illingworth et al. conducted a randomized, double-blind non-inferiority trial demonstrating that deep learning-based embryo selection achieved clinical outcomes comparable to conventional morphology-based assessment, providing important prospective evidence for the feasibility of AI-assisted embryo evaluation in routine IVF practice (66).

However, several methodological and translational limitations restrict their clinical deployment. AI model performance is highly dependent on data quality, dataset size, and population diversity, with many studies relying on single-center or retrospective cohorts. Significant data heterogeneity arises from differences in laboratory protocols, imaging systems, and culture conditions across IVF centers, limiting generalizability. In addition, the lack of standardized clinical endpoints, particularly the inconsistent use of implantation rate vs. live birth rate, further complicates model training and validation. Interoperability issues and limited external validation across independent cohorts remain major barriers to clinical adoption.

Despite encouraging results, the translation of AI assisted biomarker integration into routine clinical practice remains limited. Beyond data-driven performance, clinical implementation is constrained by issues robustness, interpretability, and regulatory requirements. Integration into existing IVF laboratory workflows further requires careful consideration of usability, standardization, and clinical governance (67).

As omic technologies and computational approaches continue to mature their combined application is expected to play an increasingly central role in personalized IVF strategies. The challenge moving forward lies in balancing technological sophistication with clinical practicality ensuring that integrated biomarker models are robust, interpretable and capable of delivering tangible improvements in patient outcomes (68). These approaches differ substantially in their level of clinical maturity, which is systematically addressed in the translational framework presented in Section 5.3.

## Clinical translation challenges and future perspectives

### Standardization and validation challenges

Despite substantial progress in the identification of non-invasive biomarkers for IVF and ART, their translation into routine clinical practice remains limited. One of the principal challenges is the lack of standardization across laboratories with respect to sample collection culture conditions, analytical platforms and data curation and interpretation. Variability in protocols can significantly influence biomarker profiles making cross study comparisons difficult and undermining reproducibility. Without harmonized and strict methodologies even biologically relevant biomarkers may fail to achieve clinical credibility (64).

### Clinical translation barriers

Another major barrier to implementation is the limited availability of large well designed prospective studies that directly link non-invasive biomarkers to clinically meaningful outcomes such as live birth rates. Many reported associations are derived from small cohorts or retrospective analyses which restrict their generalizability. In addition, the multifactorial nature of reproductive success complicates attribution of outcomes to single biomarkers emphasizing the need for integrative models rather than isolated indicators (69).

Cost effectiveness and accessibility further influence the adoption of novel biomarker technologies. Advanced omic analyses and AI based tools require specialized equipment, technical expertise and computational infrastructure which may not be readily available in all IVF settings. For widespread clinical uptake biomarker driven approaches must demonstrate clear advantages over existing methods in terms of outcome improvement efficiency or patient experience. Regulatory considerations also play a critical role as diagnostic tools must meet stringent standards of safety accuracy and clinical utility before approval (70).

### Translational stratification of non-invasive biomarkers in ART

To bridge the gap between biomarker discovery and clinical implementation, a structured conceptual framework is required to describe the level of clinical maturity and translational readiness of non-invasive biomarkers in ART. This framework categorizes non-invasive biomarkers into three tiers based on their degree of clinical validation, reproducibility, and integration into routine IVF practice. These include clinical-grade biomarkers, partially validated biomarkers, and exploratory research-stage approaches (Table 2).

**Table 2:** Conceptual classification of non-invasive biomarker approaches in ART according to their current stage of clinical implementation, representative examples, and major limitations.

Tier	Biomarker category	Examples	Clinical status	Key limitation
Tier 1	Clinical-grade methods	Time-lapse imaging, embryo morphology	Routinely used	Limited predictive power, variability
Tier 2	Partially validated biomarkers	Cell-free DNA, microRNA, proteomic/metabolomic panels	Investigational clinical use	Lack of standardization, limited prospective validation
Tier 3	Exploratory/AI/multi-omics	Multi-omics integration, AI models	Research stage	Data heterogeneity, lack of external validation

Tier 1: Clinical-grade biomarkers (Established practice)

This category includes methods that are already integrated into routine IVF workflows, primarily time-lapse imaging systems and conventional embryo morphological assessment. Although widely used, these approaches remain limited by moderate predictive accuracy and inter-observer or inter-laboratory variability. Their role is therefore primarily supportive rather than definitive in embryo selection.

Tier 2: Partially validated biomarkers (Emerging clinical tools)

This group includes circulating cell-free DNA-based approaches, microRNA signatures, and selected proteomic or metabolomic panels derived from reproductive fluids and embryo culture media. These biomarkers demonstrate reproducible associations with embryo competence, implantation potential, and pregnancy outcomes in multiple studies. However, their clinical adoption remains constrained by limited large-scale prospective validation, lack of standardized analytical pipelines, and variability across laboratories and platforms.

Tier 3: Exploratory and research-stage biomarkers (Preclinical development)

This category encompasses multi-omics integration strategies, systems biology approaches, and artificial intelligence-based predictive models. While these approaches offer high-dimensional biological insight and promising predictive performance in experimental settings, they remain predominantly confined to research environments. Key limitations include data heterogeneity, small cohort sizes, lack of external validation, and absence of standardized clinical endpoints.

Looking forward, the future of non-invasive biomarkers in IVF is closely aligned with the concept of personalized reproductive medicine. Continued refinement of biomarker discovery coupled with integrative analytical frameworks is expected to enable individualized assessment of embryo oocyte and endometrial competence. Longitudinal monitoring and adaptive treatment strategies may allow clinicians to tailor interventions to the unique biological profile of each patient. As evidence accumulates and technologies mature non-invasive biomarkers are poised to transition from experimental tools to integral components of precision driven reproductive care (36, 52).

## References

[1] Adamson GD Creighton P de Mouzon J Zegers-Hochschild F Dyer S Chambers GM. How many infants have been born with the help of assisted reproductive technology? Fertil Steril. (2025) 124:40–50. 10.1016/j.fertnstert.2025.02.009 39947276

[2] Guerif F Le Gouge A Giraudeau B Poindron J Bidault R Gasnier O. Limited value of morphological assessment at days 1 and 2 to predict blastocyst development potential: a prospective study based on 4042 embryos. Hum Reprod. (2007) 22:1973–81. 10.1093/humrep/dem100 17496054

[3] Li J Liu H Lim J Xing H Chen Y Yang S. Molecular and biological markers for assessing endometrial receptivity in infertile women: a narrative review. J Int Med Res. (2025) 53:3000605251328893. 10.1177/03000605251328893 40300557 PMC12041708

[4] Mrugacz G Mospinek A Głowacka J Sprawski O Kawałek L Gąsior W. Noninvasive preimplantation genetic testing in recurrent pregnancy loss and implantation failure: breakthrough or overpromise? Cells. (2025) 14:1591. 10.3390/cells14201591 41148806 PMC12563247

[5] Aslan Öztürk S Kiliç NÖ Kütük D Öner Ç. Fluid biopsies for *in vitro* fertilization: non-invasive innovations (review). Med Int (Lond). (2026) 6:6. 10.3892/mi.2025.290 41473679 PMC12746058

[6] Deng S Xu Y Warden AR Xu L Duan X He J. Quantitative proteomics and metabolomics of culture Medium from single human embryo reveal embryo quality-related multiomics biomarkers. Anal Chem. (2024) 96:11832–44. 10.1021/acs.analchem.4c01494 38979898

[7] Alizadeh Moghadam Masouleh A Eftekhari-Yazdi P Ebrahimi Sadrabadi A Jafarzadeh Esfehani R Tobler M Schuchardt S. Embryo metabolism as a novel non-invasive preimplantation test: nutrients turn over and metabolomic analysis of human spent embryo culture media (SECM). Hum Reprod Update. (2025) 31:405–44. 10.1093/humupd/dmaf015 40690766

[8] Mina A Younesi M Doohandeh T Darzi S Ardehjani NA Sheibani S. Predicting pregnancy outcomes in IVF cycles: a systematic review and diagnostic meta- analysis of artificial intelligence in embryo assessment. Contracept Reprod Med. (2025) 10:59. 10.1186/s40834-025-00400-4 41016945 PMC12476623

[9] Volovsky M Seifer DB. Current Status of ovarian and endometrial biomarkers in predicting ART outcomes. J Clin Med. (2024) 13:3739. 10.3390/jcm13133739 38999305 PMC11242103

[10] Rienzi L Vajta G Ubaldi F. Predictive value of oocyte morphology in human IVF: a systematic review of the literature. Hum Reprod Update. (2011) 17:34–45. 10.1093/humupd/dmq029 20639518 PMC3001337

[11] Elkhatib I Kalafat E Bayram A Abdala A Linan A Melado L. Blastulation and ploidy prediction using morphology assessment in 33,999 day-3 embryos. Sci Rep. (2025) 15:43475. 10.1038/s41598-025-19898-4 41365919 PMC12690125

[12] Jiang X Cai J Liu L Liu Z Wang W Chen J. Does conventional morphological evaluation still play a role in predicting blastocyst formation? Reprod Biol Endocrinol. (2022) 20:68. 10.1186/s12958-022-00945-y 35439999 PMC9016972

[13] Chen K Hu Z Lian Y Han Y Zhou X Li Y. The diagnostic accuracy of preimplantation genetic testing (PGT) in assessing the genetic status of embryos: a systematic review and meta-analysis. Reprod Biol Endocrinol. (2025) 23:39. 10.1186/s12958-025-01376-1 40069837 PMC11895315

[14] Bouba I Hatzi E Ladias P Sakaloglou P Kostoulas C Georgiou I. Biological and clinical significance of mosaicism in human preimplantation embryos. J Dev Biol. (2021) 9:18. 10.3390/jdb9020018 34066950 PMC8162329

[15] Liu Z Liu X Wang M Zhao H He S Lai S. The clinical efficacy of personalized embryo transfer guided by the endometrial receptivity array/analysis on IVF/ICSI outcomes: a systematic review and meta-analysis. Front Physiol. (2022) 13:841437. 10.3389/fphys.2022.841437 35574479 PMC9092494

[16] Mrugacz G Bołkun I Magoń T Korowaj I Golka B Pluta T. Time-Lapse imaging in IVF: bridging the gap between promises and clinical realities. Int J Mol Sci. (2025) 26:9609. 10.3390/ijms26199609 41096871 PMC12524532

[17] Dare MR Crowther CA Dodd JM Norman RJ. Single or multiple embryo transfer following *in vitro* fertilisation for improved neonatal outcome: a systematic review of the literature. Aust N Z J Obstet Gynaecol. (2004) 44:283–91. 10.1111/j.1479-828X.2004.00243.x 15281996

[18] Tesarik J. Noninvasive biomarkers of human embryo developmental potential. Int J Mol Sci. (2025) 26:4928. 10.3390/ijms26104928 40430065 PMC12112732

[19] Palomar A Yagüe-Serrano R Martínez-Sanchis JV Iniesta I Quiñonero A Fernández-Colom PJ. Predictive potential of combined secretomics and image-based morphometry as a non-invasive method for selecting implanting embryos. Reprod Biol Endocrinol. (2025) 23:57. 10.1186/s12958-025-01386-z 40221726 PMC11992772

[20] Giménez C Conversa L Murria L Meseguer M. Time-lapse imaging: morphokinetic analysis of *in vitro* fertilization outcomes. Fertil Steril. (2023) 120:218–27. 10.1016/j.fertnstert.2023.06.015 37364673

[21] Sayed S Reigstad MM Petersen BM Schwennicke A Wegner Hausken J Storeng R. Time-lapse imaging derived morphokinetic variables reveal association with implantation and live birth following *in vitro* fertilization: a retrospective study using data from transferred human embryos. PLoS One. (2020) 15:e0242377. 10.1371/journal.pone.0242377 33211770 PMC7676704

[22] Maghiar L Naghi P Zaha IA Sandor M Bodog A Sachelarie L. Correlation between human embryo morphokinetics observed through time-lapse incubator and life birth rate. J Pers Med. (2024) 14:1045. 10.3390/jpm14101045 39452552 PMC11508429

[23] Bhide P Chan DYL Lanz D Alqawasmeh O Barry E Baxter D. Clinical effectiveness and safety of time-lapse imaging systems for embryo incubation and selection in *in-vitro* fertilisation treatment (TILT): a multicentre, three-parallel-group, double-blind, randomised controlled trial. Lancet. (2024) 404:256–65. 10.1016/S0140-6736(24)00816-X 39033010

[24] Armstrong S Bhide P Jordan V Pacey A Marjoribanks J Farquhar C. Time-lapse systems for embryo incubation and assessment in assisted reproduction. Cochrane Database Syst Rev. (2019) 5:CD011320. 10.1002/14651858.CD011320.pub4 31140578 PMC6539473

[25] Kieslinger DC Vergouw CG Ramos L Arends B Curfs MHJM Slappendel E. Clinical outcomes of uninterrupted embryo culture with or without time-lapse-based embryo selection versus interrupted standard culture (SelecTIMO): a three-armed, multicentre, double-blind, randomised controlled trial. Lancet. (2023) 401:1438–46. 10.1016/S0140-6736(23)00168-X 37004670

[26] Cabello-Pinedo S Abdulla H Mas S Fraire A Maroto B Seth-Smith M. Development of a novel non-invasive metabolomics assay to predict implantation potential of human embryos. Reprod Sci. (2024) 31:2706–17. 10.1007/s43032-024-01583-y 38834841

[27] Toporcerová S Badovská Z Kriváková E Mikulová V Mareková M Altmäe S. Embryo secretome in predicting embryo quality and IVF treatment outcome. Reprod Biomed Online. (2025) 51:104825. 10.1016/j.rbmo.2025.104825 40446645

[28] Thuwanut P Sirayapiwat P Roytrakul S Tuntiviriyapun P Suebthawinkul C Panyawongudom N. Peptidomic and proteomic signatures in human blood Serum, follicular fluid and spent Media: a study of embryo development competency after *in vitro* fertilization. Reprod Sci. (2025) 32:2654–68. 10.1007/s43032-025-01933-4 40665108

[29] Rosenbluth EM Shelton DN Wells LM Sparks AET Van Voorhis BJ. Human embryos secrete microRNAs into culture media–a potential biomarker for implantation. Fertil Steril. (2014) 101:1493–500. 10.1016/j.fertnstert.2014.01.058 24786747

[30] Capalbo A Ubaldi FM Cimadomo D Noli L Khalaf Y Farcomeni A. MicroRNAs in spent blastocyst culture medium are derived from trophectoderm cells and can be explored for human embryo reproductive competence assessment. Fertil Steril. (2016) 105:225–235.e1–3. 10.1016/j.fertnstert.2015.09.014 26453979

[31] Zhou W Dimitriadis E. Secreted MicroRNA to predict embryo implantation outcome: from research to clinical diagnostic application. Front Cell Dev Biol. (2020) 8:586510. 10.3389/fcell.2020.586510 33072767 PMC7537741

[32] Sialakouma A Karakasiliotis I Ntala V Nikolettos N Asimakopoulos B. Embryonic cell-free DNA in spent culture Medium: a non-invasive tool for aneuploidy screening of the corresponding embryos. In Vivo. (2021) 35:3449–57. 10.21873/invivo.12645 34697181 PMC8627731

[33] Handayani N Aubry D Boediono A Bowolaksono A Sini I Haq NMD. Non-invasive pre-implantation genetic testing’s reliability for aneuploidy using cell-free DNA in embryo culture media. J Gynecol Obstet Hum Reprod. (2024) 53:102808. 10.1016/j.jogoh.2024.102808 38825167

[34] Yang S Xu B Zhuang Y Zhang Q Li J Fu X. Current research status and clinical applications of noninvasive preimplantation genetic testing: a review. Medicine (Baltimore). (2024) 103:e39964. 10.1097/MD.0000000000039964 39465745 PMC11460858

[35] Tomic M Vrtacnik Bokal E Stimpfel M. Non-Invasive preimplantation genetic testing for aneuploidy and the mystery of genetic material: a review article. Int J Mol Sci. (2022) 23:3568. 10.3390/ijms23073568 35408927 PMC8998436

[36] Farzizadeh N Amoozgar M Aminbeidokhti M Hariri A Khosravi A Najmi Z. Non-invasive biomarkers in assisted reproduction: a comprehensive review for predicting IVF success. J Assist Reprod Genet. (2026) 43:35–50. 10.1007/s10815-025-03707-y 41171499 PMC12831755

[37] Pan Y Pan C Zhang C. Unraveling the complexity of follicular fluid: insights into its composition, function, and clinical implications. J Ovarian Res. (2024) 17:237. 10.1186/s13048-024-01551-9 39593094 PMC11590415

[38] Albeitawi S Bani-Mousa S-U Jarrar B Aloqaily I Al-Shlool N Alsheyab G. Associations between follicular fluid biomarkers and IVF/ICSI outcomes in normo-ovulatory women-A. Systematic Review. Biomolecules. (2025) 15:443. 10.3390/biom15030443 40149979 PMC11940193

[39] Chen F Spiessens C D’Hooghe T Peeraer K Carpentier S. Follicular fluid biomarkers for human *in vitro* fertilization outcome: proof of principle. Proteome Sci. (2016) 14:17. 10.1186/s12953-016-0106-9 27895531 PMC5109724

[40] Lv Y Du S Huang X Hao C. Follicular fluid estradiol is an improved predictor of *in vitro* fertilization/intracytoplasmic sperm injection and embryo transfer outcomes. Exp Ther Med. (2020) 20:131. 10.3892/etm.2020.9256 33082863 PMC7557525

[41] Pizarro BM Cordeiro A Reginatto MW Campos SPC Mancebo ACA Areas PCF. Estradiol and progesterone levels are related to redox Status in the follicular fluid during *in vitro* fertilization. J Endocr Soc. (2020) 4:bvaa064. 10.1210/jendso/bvaa064 32666010 PMC7326473

[42] Jiang YC Che Q Lu X Liu M Ye Y Cao X. Follicular fluid and plasma lipidome profiling and associations towards embryonic development outcomes during ART treatment. Front Endocrinol (Lausanne). (2024) 15:1464171. 10.3389/fendo.2024.1464171 39790287 PMC11712041

[43] Hood RB Liang D Tan Y Ford JB Souter I Chavarro JE. Serum and follicular fluid metabolome and markers of ovarian stimulation. Hum Reprod. (2023) 38:2196–207. 10.1093/humrep/dead189 37740688 PMC10628502

[44] Stokes AE Clark HM Edwards JL Payton RR Beever JE Freeman TF. Transcriptome profiles of blastocysts originating from oocytes matured in follicular fluid from preovulatory follicles of greater or lesser maturity. BMC Genomics. (2025) 26:339. 10.1186/s12864-025-11521-0 40186098 PMC11969919

[45] Voros C Athanasiou D Papapanagiotou I Mavrogianni D Varthaliti A Bananis K. Cracking the code of oocyte quality: the oxidative stress link to IVF success. Int J Mol Sci. (2025) 26:6377. 10.3390/ijms26136377 40650156 PMC12249650

[46] Terao H Wada-Hiraike O Nagumo A Kunitomi C Azhary JMK Harada M. Role of oxidative stress in follicular fluid on embryos of patients undergoing assisted reproductive technology treatment. J Obstet Gynaecol Res. (2019) 45:1884–91. 10.1111/jog.14040 31257684

[47] Zhang X Zhang L Xiang W. The impact of mitochondrial dysfunction on ovarian aging. J Transl Med. (2025) 23:211. 10.1186/s12967-025-06223-w 39980008 PMC11844166

[48] Wang S Ren J Jing Y Qu J Liu G-H. Perspectives on biomarkers of reproductive aging for fertility and beyond. Nat Aging. (2024) 4:1697–710. 10.1038/s43587-024-00770-5 39672897

[49] Dai M Hong L Yin T Liu S. Disturbed follicular microenvironment in polycystic ovary syndrome: relationship to oocyte quality and infertility. Endocrinology. (2024) 165:bqae023. 10.1210/endocr/bqae023 38375912

[50] Han M-T Cheng W Zhu R Wu H-H Ding J Zhao N-N. The cytokine profiles in follicular fluid and reproductive outcomes in women with endometriosis. Am J Reprod Immunol. (2023) 89:e13633. 10.1111/aji.13633 36250899

[51] Stojanovic Gavrilovic AZ Cekovic JM Parandilovic AZ Nikolov AB Sazdanovic PS Velickovic AM. IL-6 of follicular fluid and outcome of *in vitro* fertilization. Medicine (Baltimore). (2022) 101:e29624. 10.1097/MD.0000000000029624 35866786 PMC9302246

[52] Voros C Varthaliti A Athanasiou D Mavrogianni D Bananis K Athanasiou A. MicroRNA signatures in endometrial receptivity-unlocking their role in embryo implantation and IVF success: a systematic review. Biomedicines. (2025) 13:1189. 10.3390/biomedicines13051189 40427016 PMC12109305

[53] Zeng H Chang Y Fu J Tang H Liu N Luo B. Inflammatory proteomics of uterine fluid is potentially a non-invasive predictor for endometrial receptivity: a pilot study. J Assist Reprod Genet. (2026) 43:307–16. 10.1007/s10815-025-03728-7 41175188 PMC12831789

[54] Apostolov A Mladenović D Tilk K Lõhmus A Baev V Yahubyan G. Multi-omics analysis of uterine fluid extracellular vesicles reveals a resemblance with endometrial tissue across the menstrual cycle: biological and translational insights. Hum Reprod Open. (2025) 2025:hoaf010. 10.1093/hropen/hoaf010 40084293 PMC11904304

[55] Marzanati D Ranucci F Pagliardini L Vanni VS Barberis N Papaleo E. Endometrial receptivity profiled through transcriptomic analysis of uterine fluid extracellular vesicles using systems biology and Bayesian modeling for pregnancy prediction. Sci Rep. (2025) 15:35634. 10.1038/s41598-025-19633-z 41083775 PMC12518540

[56] Alves AR Dias MF Silvestre M. Endometrial fluid biomarkers and their potential as predictors of successful embryo implantation. Biomedicine (Taipei). (2023) 13:1–8. 10.37796/2211-8039.1413 37937060 PMC10627212

[57] Wu C Sun Y Yang D Peng H. Advances in endometrial receptivity and embryo implantation by multi-omics techniques. Animals and Zoonoses. (2025) 1:286–94. 10.1016/j.azn.2025.05.001

[58] Karadbhajne P More A Dzoagbe HY. The role of endometrial Microbiota in fertility and reproductive health: a narrative review. Cureus. (2025) 17:e78982. 10.7759/cureus.78982 40099081 PMC11911285

[59] Stoyancheva G Mihaylova N Gerginova M Krumova E. Endometrial microbiome and reproductive receptivity: diverse perspectives. Int J Mol Sci. (2025) 26:10796. 10.3390/ijms262110796 41226831 PMC12609489

[60] Jin Q Zhao J Ma Y Zhang Y Yan M Song J. Integrated proteomic and metabolomic analysis elucidates the effects and mechanisms of qiziyusi decoction on IVF outcomes in advanced maternal age infertility. Front Endocrinol (Lausanne). (2025) 16:1573206. 10.3389/fendo.2025.1573206 41141348 PMC12549270

[61] Baião AR Cai Z Poulos RC Robinson PJ Reddel RR Zhong Q. A technical review of multi-omics data integration methods: from classical statistical to deep generative approaches. Brief Bioinform. (2025) 26:bbaf355. 10.1093/bib/bbaf355 40748323 PMC12315550

[62] Ou Y Wang H Zhou C Chen Y Lyu J Feng M. Endometriosis-associated infertility: multi-omics insights into pathogenesis and precision therapeutics. Front Endocrinol (Lausanne). (2025) 16:1613334. 10.3389/fendo.2025.1613334 41113721 PMC12527851

[63] AlSaad R Abd-Alrazaq A Choucair F Ahmed A Aziz S Sheikh J. Harnessing artificial intelligence to predict ovarian stimulation outcomes in *in vitro* fertilization: scoping review. J Med Internet Res. (2024) 26:e53396. 10.2196/53396 38967964 PMC11259766

[64] Gao Y Yuan Y Wang K Wang Y Gao T Yang Y. Current progress and open challenges for applying artificial intelligence across the *in vitro* fertilization cycle. Patterns (N Y). (2025) 6:101347. 10.1016/j.patter.2025.101347 41328159 PMC12664965

[65] Khosravi P Kazemi E Zhan Q Malmsten JE Toschi M Zisimopoulos P. Deep learning enables robust assessment and selection of human blastocysts after *in vitro* fertilization. NPJ Digit Med. (2019) 2:21. 10.1038/s41746-019-0096-y 31304368 PMC6550169

[66] Illingworth PJ Venetis C Gardner DK Nelson SM Berntsen J Larman MG. Deep learning versus manual morphology-based embryo selection in IVF: a randomized, double-blind noninferiority trial. Nat Med. (2024) 30:3114–20. 10.1038/s41591-024-03166-5 39122964 PMC11564097

[67] Thirumalaraju P Kanakasabapathy MK Kandula H Kandula T Reddy Katkuri AV Cipriano C. Stability and reliability of artificial intelligence models in embryo selection for *in vitro* fertilization. Fertil Steril. (2025) S0015-0282(25):01845–X. 10.1016/j.fertnstert.2025.08.021 PMC12494150 40876725

[68] Mohr AE Ortega-Santos CP Whisner CM Klein-Seetharaman J Jasbi P. Navigating challenges and opportunities in multi-omics integration for personalized healthcare. Biomedicines. (2024) 12:1496. 10.3390/biomedicines12071496 39062068 PMC11274472

[69] Šmak P Juřica J Gregorová J Porokh V Peš O Holubcová Z. The quest for metabolic biomarkers of IVF outcomes: a meta-analysis and critical review. Front Cell Dev Biol. (2025) 13:1640807. 10.3389/fcell.2025.1640807 41181703 PMC12575374

[70] Mrugacz G Mospinek A Jagielska M Miszczak D Matosek A Ducher-Hanaka M. Artificial intelligence in routine IVF practice. Biology (Basel). (2025) 15:42. 10.3390/biology15010042 41514884 PMC12784763

